# A comparative study of different antiviral treatment protocols in HCV related cryoglobulinemic vasculitis

**DOI:** 10.1038/s41598-024-60490-z

**Published:** 2024-05-23

**Authors:** Walaa Ramadan Allam, Mohamed Tharwat Hegazy, Mohamed A. Hussein, Naguib Zoheir, Luca Quartuccio, Sherif F. El-Khamisy, Gaafar Ragab

**Affiliations:** 1 Center for Genomics, Zewail City of Science and Technology, Giza, Egypt; 2 Internal Medicine Department, Rheumatology and Clinical Immunology Unit, Faculty of Medicine, Cairo University, Cairo, Egypt; 3 School of Medicine, Newgiza University (NGU), Giza, Egypt; 4 Clinical and Chemical Pathology Department, Faculty of Medicine, Cairo University, Cairo, Egypt; 5 Clinic of Rheumatology, Department of Medical Area (DAME), University Hospital “Santa Maria Della Misericordia”, University of Udine, Udine, Italy; 6 The Healthy Lifespan and the Institute of Neuroscience, University of Sheffield, Sheffield, S10 2TN UK

**Keywords:** Directly acting antiviral drugs (DAAs), HCV-induced mixed cryoglobulinemic vasculitis (HCV–MCV), Interferon, Genomic instability, DNA damage, DNA repair, BAFF, APRIL, Gastroenterology, Medical research, Rheumatology

## Abstract

The treatment of HCV and its sequelae are used to be predominantly based on Interferon (IFN). However, this was associated with significant adverse events as a result of its immunostimulant capabilities. Since their introduction, the directly acting antiviral drugs (DAAs), have become the standard of care to treat of HCV and its complications including mixed cryoglobulinemic vasculitis (MCV). In spite of achieving sustained viral response (SVR), there appeared many reports describing unwelcome complications such as hepatocellular and hematological malignancies as well as relapses. Prolonged inflammation induced by a multitude of factors, can lead to DNA damage and affects BAFF and APRIL, which serve as markers of B-cell proliferation. We compared, head-to-head, three antiviral protocols for HCV–MCV treatment As regards the treatment response and relapse, levels of BAFF and APRIL among pegylated interferon α-based and free regimens (Sofosbuvir + Ribavirin; SOF–RIBA, Sofosbuvir + Daclatasvir; SOF–DACLA). Regarding clinical response HCV–MCV and SVR; no significant differences could be identified among the 3 different treatment protocols, and this was also independent form using IFN. We found no significant differences between IFN-based and free regimens DNA damage, markers of DNA repair, or levels of BAFF and APRIL. However, individualized drug-to-drug comparisons showed many differences. Those who were treated with IFN-based protocol showed decreased levels of DNA damage, while the other two IFN-free groups showed increased DNA damage, being the worst in SOF–DACLA group. There were increased levels of BAFF through follow-up periods in the 3 protocols being the best in SOF–DACLA group (decreased at 24 weeks). In SOF–RIBA, CGs relapsed significantly during the follow-up period. None of our patients who were treated with IFN-based protocol had significant clinico-laboratory relapse. Those who received IFN-free DAAs showed a statistically significant relapse of constitutional manifestations. Our findings suggest that IFN-based protocols are effective in treating HCV–MCV similar to IFN-free protocols. They showed lower levels of DNA damage and repair. We believe that our findings may offer an explanation for the process of lymphoproliferation, occurrence of malignancies, and relapses by shedding light on such possible mechanisms.

## Introduction

Hepatitis C virus can result in many hepatic and extrahepatic manifestations which culminate in significant morbidity and mortality. Before the introduction of directly acting antiviral drugs (DAAs), the treatment of HCV was based predominantly on Interferon (IFN). The drug proved partially successful, but its use was associated with significant adverse events, as a result of its immunostimulant capabilities. This carried the risk of provoking autoimmunity with hazardous sequelae which led to the negative outlook on this line of therapy^1,2^.

Since their introduction in 2013, DAAs have become the standard of care for the treatment of HCV and its related complications including mixed cryoglobulinemic vasculitis (MCV)^3^. However, following their great promise, some reports appeared describing hepatocellular and hematological malignancies despite sustained viral response (SVR)^4–7^.

Prolonged inflammation induced by a multitude of factors can lead to DNA damage and activate the DNA damage/repair process. This can be caused by infections, autoimmunity, or medications^8–10^.

HCV has also been shown to induce many B-cell lymphoproliferative disorders^11^. BAFF and APRIL serve as markers of B-cell proliferation^12^, hence they can be used for this purpose in HCV–MCV, being B-cell attributed disorder^11^, to evaluate the risk of development or disappearance after successful HCV treatment.

Relapse of MCV has been described in many reports where BAFF and April are hypothesized to play a role in the relapses of MCV and the possible development of lymphoid neoplasm^13,14^.

Colantuono and his colleagues recommend the pivotal need for a better understanding of the mechanisms underlying HCV–MCV resistance to the cure as well as identifying patients at risk and the possibility to avoid relapses^12^.

The findings in all previous workers support the notion that immunosuppressive drugs need to be added to antiviral protocols^11^ targeting B cells or BAFF and APRIL^15^.

In a previous study, we documented increased genomic instability markers and we also reported changes in BAFF and APRIL levels induced by different DAAs protocols regardless of the treatment protocol^16^.

Motivated by the observations on the course of HCV–MCV following SVR, the emergence of HCC and lymphoma as well as relapse, we revisited the data from our previous study^16^ and carried a head-to-head comparison between different antiviral protocols for the treatment of HCV–MCV. We compared pegylated interferon α (pIFN α) based and free regimens as well as shorter “12 weeks” and longer “24 weeks” duration treatment protocols. We studied treatment response as well as DNA damage and repair among differently used treatment protocols.

We explored the impact of interferon-based drugs in particular on the immune system and their effect on BAFF and APRIL since this had been observed in the era preceding the direct-acting antiviral drugs.

### Patients and methods

Our study was an observational analytical study. Our data were reported from HCV patients who received anti-HCV medications according to the protocols approved and endorsed by the Egyptian Ministry of Health (MOH) during the National campaign to eradicate HCV. We planned to study the outcome of HCV eradication on HCV–MCV as a subgroup of patients. The outcome was reported collectively in previous publications^16–18^ but in the current study we compare the different protocols regarding treatment response and relapse as well as DNA damage and repair among differently protocols.

The study was conducted in the National Hepatology and Tropical Medicine Research Institute and followed the ethical standards of the National Research Committee and the 1964 Helsinki Declaration and its later amendments and was approved by the local institutional research board as well as the institutional review board for human subject research at The National Hepatology and Tropical Medicine Research Institute (NHTMRI) (IRB: 20-2016). Informed consent was obtained from all included patients.

This interventional study included 32 Egyptian patients with HCV–MCV diagnosed according to the validated 2014 classification criteria of cryoglobulinemic vasculitis (CV)^19^. Patients received three different antiviral protocols approved by the Egyptian Ministry of Health during the period of study (2014–2017). Group (1) received IFN based while groups 2 and 3 received IFN-free protocols. Group (1) Included 8 patients who received pIFN plus Sofosbuvir (SOF) and ribavirin (RBV) for 12 weeks. Group (2) included 13 patients treated with SOF plus RBV for 24 weeks and group (3) included 11 patients who received SOF plus Daclatasvir (DACLA) for 12 weeks.

Immunosuppressant usage was limited to oral prednisolone at a maximum dose of 30 mg/day. A washout period of at least 4 weeks was needed for higher doses of steroids and other immunosuppressants except rituximab (where it reached 6 months).

Clinical complete response (CR) referred to complete recovery while partial response (PR) was defined as a decrease of ≥ 50% of clinical manifestations “compared with baseline”. Those with neither CR nor PR were classified as non-responders (NR).

With the absence of specific renal response criteria in MCV, we applied the American College of Rheumatology (ACR) renal response criteria in systemic lupus erythematosus (SLE)^20^ to assess renal response in our patients.

Normalization of serum rheumatoid factor (RF) and complement 4 (C4) levels and disappearance of circulating cryoglobulins (CGs) referred to laboratory CR while ≥ 50% decrease “compared with baseline” means PR. Those who didn’t fulfill CR or PR were classified as NR.

Relapse was defined as worsening or reappearance of clinical and/or laboratory markers at one year follow-up compared to the end of treatment (EOT).

Serum RF and C4 were assayed by nephelometry using BN ProSpec; Siemens, Germany. RF is positive if > 15 IU/ml, C4 is consumed if < 10 mg/dl). The CGs were obtained by cold precipitation (at 4˚C for 1 week) considered positive if more than 1%.

The Liver fibrosis was evaluated by abdominal ultrasound and Fibro scan.

### Quantification of Double Strands breaks (DSBs) in patient's peripheral blood mononuclear cells (PBMCs)

PBMCs were isolated from fresh whole blood samples using the Ficoll-Hypaque density gradient centrifugation method, and DSBs were quantified by neutral comet assay as described in^21^.

### Quantification of gene expression assays using taqman probe assays

The cDNA product from the RT reaction was used for Taqman PCR quantification in a final reaction volume of 20 μl, using the Sensi FAST Probe low-ROX master mix (BIOLINE, UK). A 20 × mix of primers and FAM-labeled probe for the human TOP1, TOP2B, TDP1, TDP2, PARP1, XRCC1, APRIL, and BAFF gene expression assays were purchased through ABI's Gene Expression Assay-on-Demand. Gene expression values were calculated using the comparative delta CT method, according to^22^.

### Statistical methods

Data were coded and entered using the statistical package SPSS (Statistical Package for the Social Sciences) version 24. Data was summarized using mean, standard deviation, median, minimum, and maximum in quantitative data and using frequency (count) and relative frequency (%) for categorical data. For the comparison of serial measurements for each patient, the non-parametric Wilcoxon signed-rank test was used^6^. For comparing categorical data, the Chi-square (χ2) test was performed. Exact test was used instead when the expected frequency was less than 5^23^. Correlations between quantitative variables used Spearman correlation coefficient^24^. *P*-values < 0.05 were considered statistically significant. Pretreatment treatment and follow-up values were analyzed and compared using paired samples t-test, at 95% confidence intervals, were calculated using the exact formula.

All analyses and graphs were performed with Graph Pad Prism version 7 (GraphPad Software, La Jolla California USA).

## Results

The study included 25 females (78.1%) and 7 males (21.9%) with a mean age of (54.9 ± 9.7) years. 14 patients had liver cirrhosis (43.8%); all of whom were Child A class with no history of previous treatment for HCV. All our patients (100%) showed negative PCR for HCV after one month of treatment and throughout the follow-up period.

**Figure 1:**
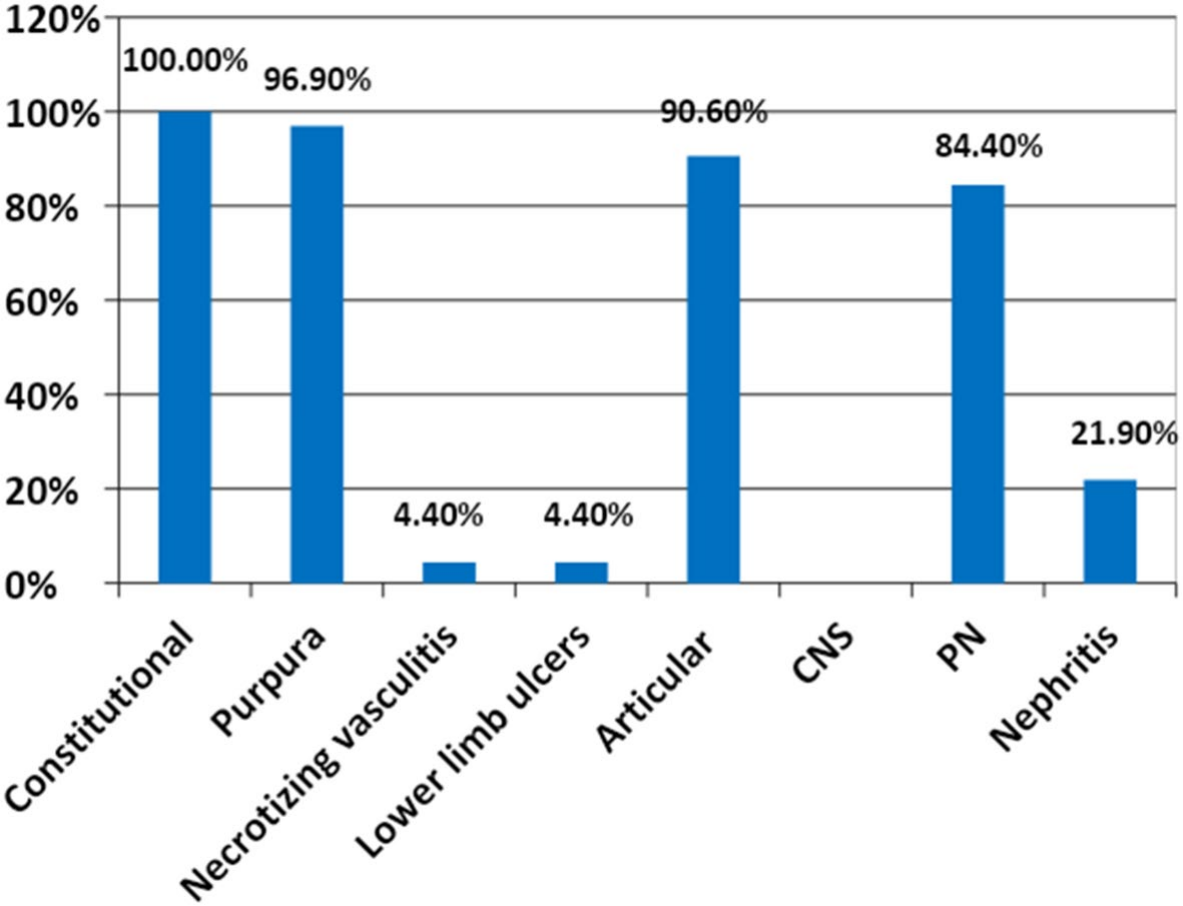
Clinical characteristics of the patients.

The clinical characteristics of our patients are shown in Fig. 1.

### Clinical and Laboratory Responses

Purpura responded in all patients (100%) in different protocols of treatment. Successful response to treatment by antiviral therapy using Birmingham vasculitis activity score 3 (BVAS 3) was 100% (group 1), 76.9% (group 2), and 90.9% (group 3), (*P* value = 0.4). Serum CGs levels improved (decreased) at EOT but not during long-term follow-up period in the three protocols**.**

#### DNA-repair genes expression profile

**Figure 2:**
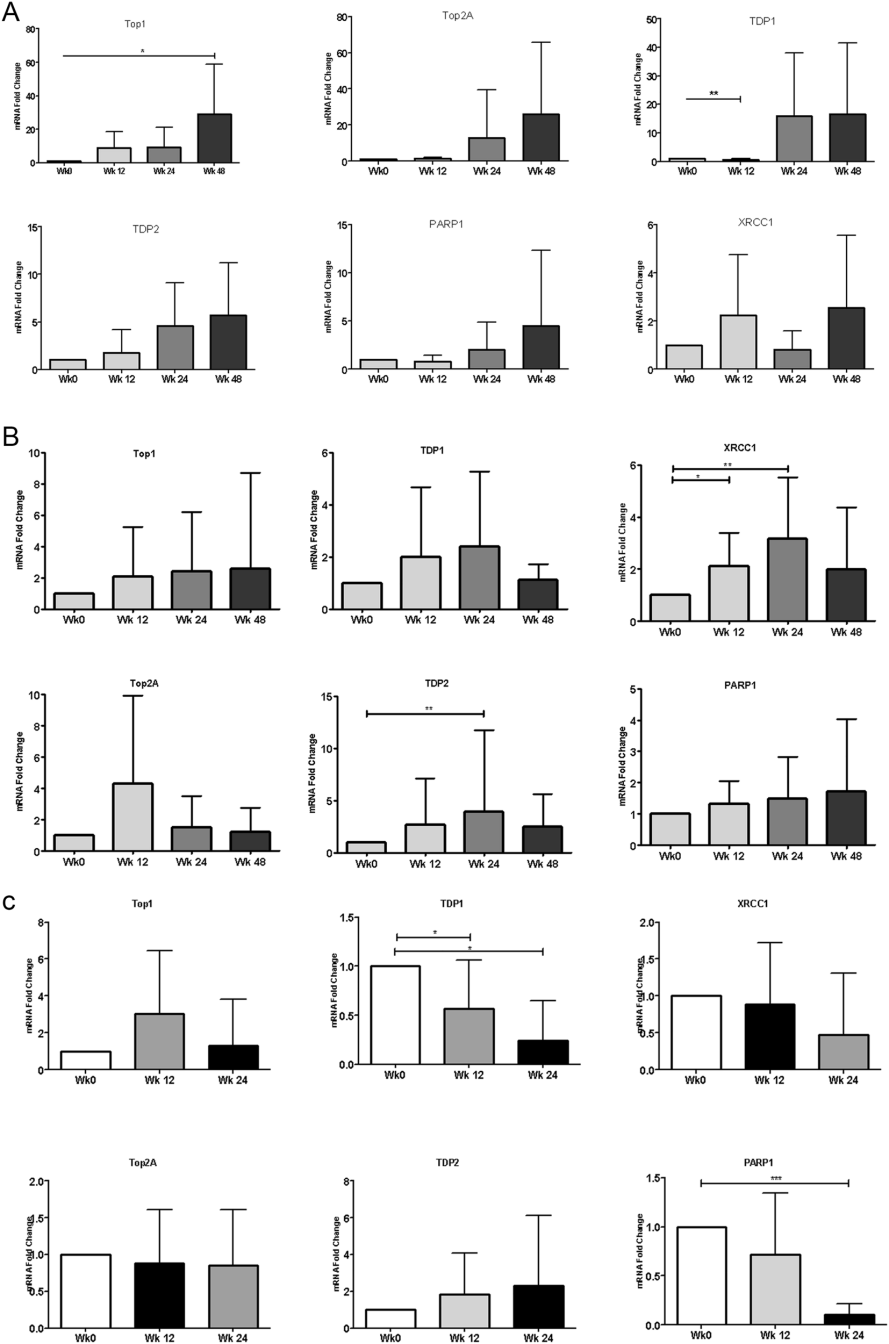
(**a**) DNA-repair genes expression profile in group (1). (b) DNA-repair genes expression profile in Group (2). (**c**) DNA-repair genes expression profile in Group (3).

A continuous increase in the expression of selected DNA repair genes was noticed in the three different regimens. In group 1, expression levels of DNA repair genes persisted until wk48 (Fig. 2a). Patients in the other two regimens showed an increase in instability markers at WK12 but expression levels showed inconsistent increase/decrease across different markers (Fig. 2b,c).

#### Gene expression profile of B cells activation factors

**Figure 3:**
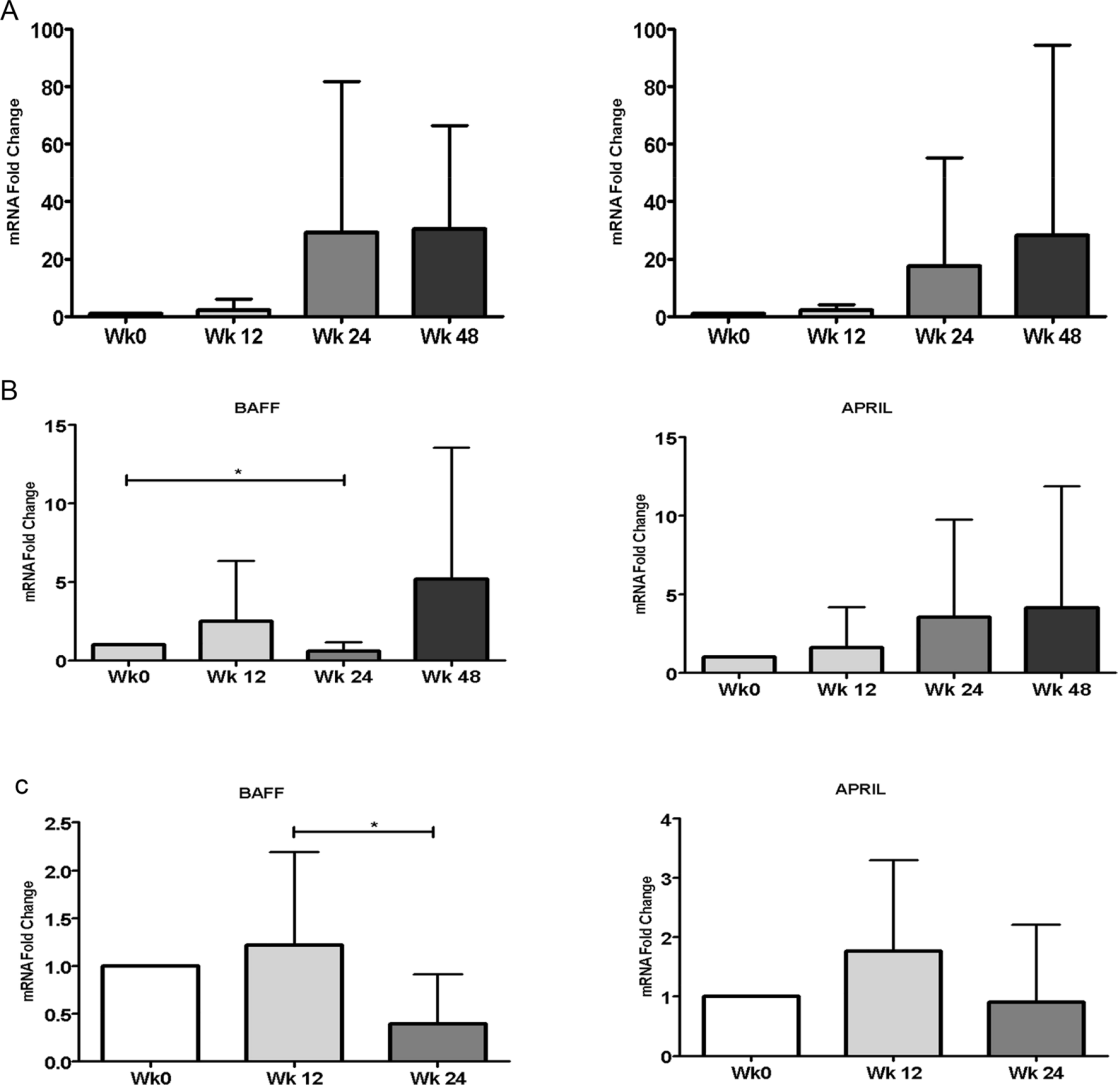
Gene expression of B cells activation factors in: Group 1 (**a**), 2 (**b**) and 3 (**c**).

The expression levels of both BAFF and APRIL increased upon starting the treatment at WK12 and maintained high levels at all follow-up points across different treatment regimens until WK 48 in groups 1 and 3 (Fig. 3a,c). In group 2, a significant decrease in B cell activation levels was noticed at a single point at WK24 (Fig. 3b). The persistent increase of B-cell activation seems to be independent of HCV infection since all the patients achieved viral eradication as early as one month from starting treatment and it might be linked to MCV relapse observed in some patients.

#### Chromosomal breaks accumulation

**Figure 4:**
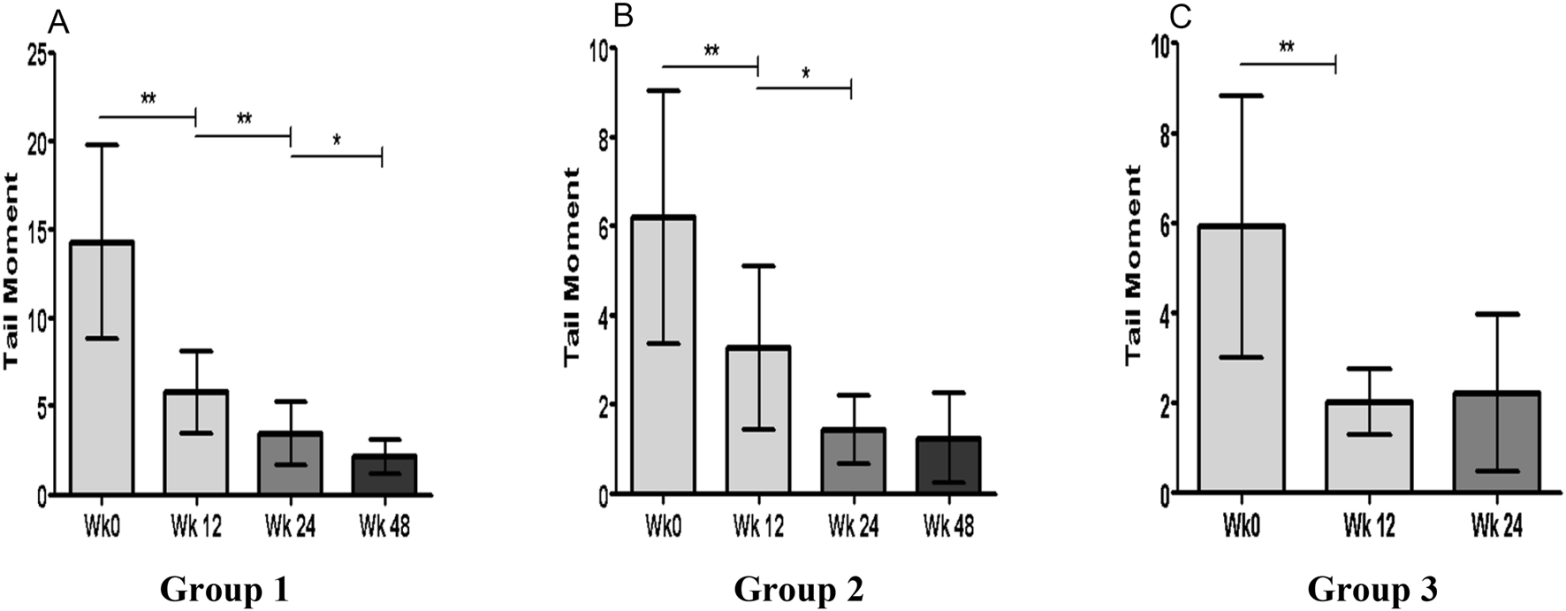
Chromosomal breaks accumulation.

A significant initial increase in DNA damage levels at WK12 was observed in all groups (P < 0.05). DNA damage levels continued to decrease at the following follow-up points to reach the lowest levels at WK 48 except in group 3 where DNA damage levels maintained similar levels at WK12 and WK24 (Fig. 4).

### IFN-based versus IFN-free protocols

Overall clinical responses of IFN-based and free regimens were (93% and 85%) and laboratory responses (78% and 84%) respectively. IFN-based regimen showed the same cutaneous response as IFN-free regimens with better articular, renal, and neurological responses, though non-significant. Clinico-laboratory response and relapse of IFN-based and free regimens are mentioned in Table 1.

**Table 1:** Clinico-laboratory response and relapse of IFN-based and free regimens.

	EOT					Relapse					
	IFN based		IFN-free		*P* value	IFN-based		*P* value	IFN-free		*P* value
	Total (N)	Response N (%)	Total (N)	Response N(%)		Total (N)	Deterioration N (%)		Total (N)	Deterioration N (%)	
Purpura (N: 31)	7	7 (100%)	24	24 (100%)	–	7	1 (14.3%)	–	15	0 (0%)	–
Articular manifestations (N:29)	7	6 (85.7%)	22	18 (81.8%)	1	6	1 (16.7%)	0.286	11	4 (36.4%)	0.192
Peripheral neuropathy (N:27)	6	6 (100%)	21	16 (76.2%)	0.555	6	3 (50%)	–	9	3 (33.3%)	1
Constitutional manifestations (N:32)	8	7 (87.5%)	24	20 (83.3%)	1	7	2 (28.6%)	0.125	12	3 (25%)	0.044
24h urinary protein (N:7)	2	2 (100%)	5	4 (80%)	1	2	1 (50)%	–	2	0 (0%)	-
Serum RF (IU/ml) (N:32)	8	7 (87.5%)	24	19 (79.2%)	1	7	2 (28.6%)	1	11	3 (27.3%)	1
Serum C4 (mg/dl) (N:32)	8	4 (50%)	24	19 (79.2%)	0.176	4	0 (0%)	1	13	2 (15.4%)	0.371
Serum CGs (%) (N:32)	8	6 (75%)	24	23 (95.8%)	0.147	6	1 (16.7%)	0.214	14	4 (28.6%)	0.067

EOT, end of therapy; IFN, interferon; PN, peripheral neuropathy; BVAS, Birmingham vasculitis activity score; RF, rheumatoid factor; C4, Complement 4; CGs, cryoglobulins.

#### DNA repair genes and B-cells activation markers expression profile

**Figure 5:**
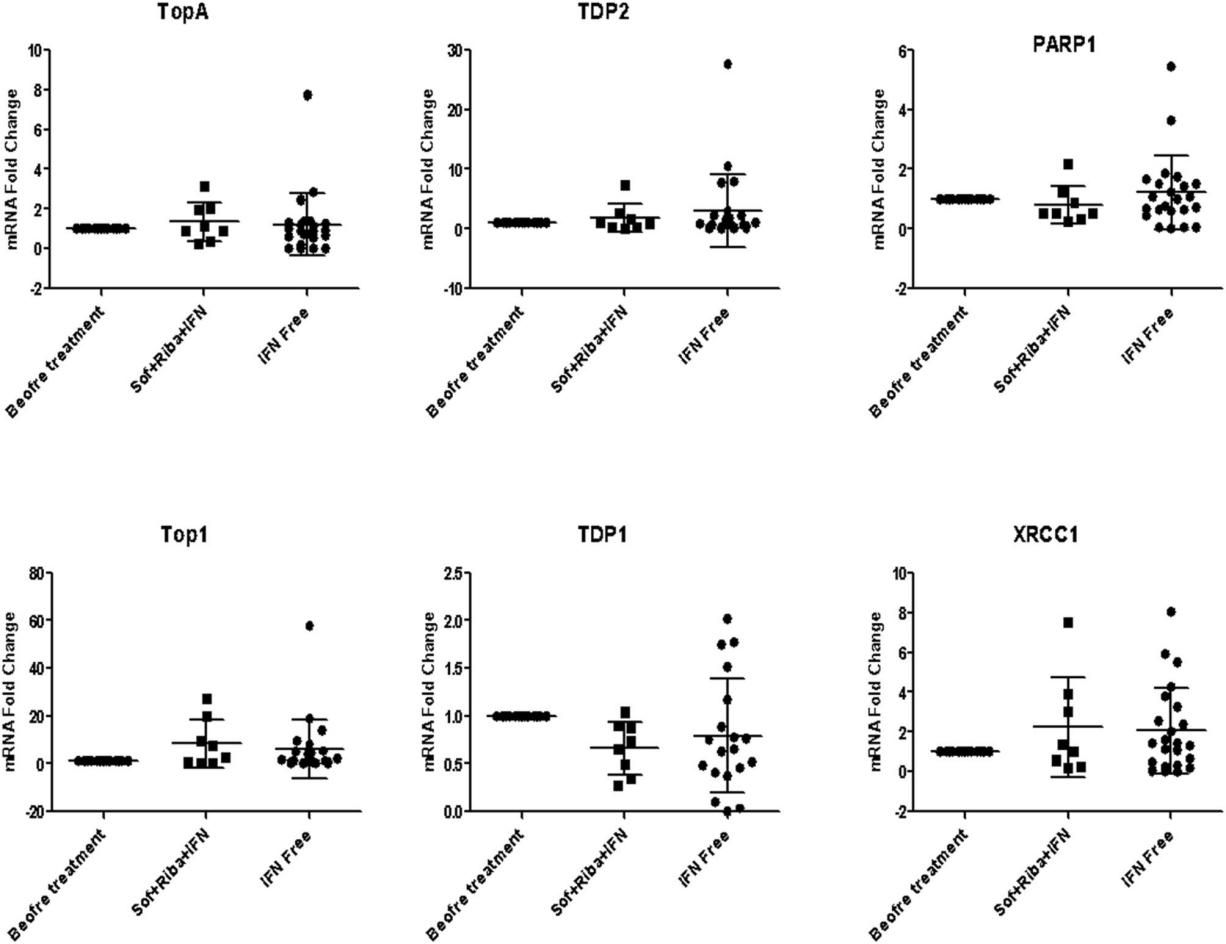
DNA repair genes expression profile in IFN-based and IFN-free protocols.

**Figure 6:**
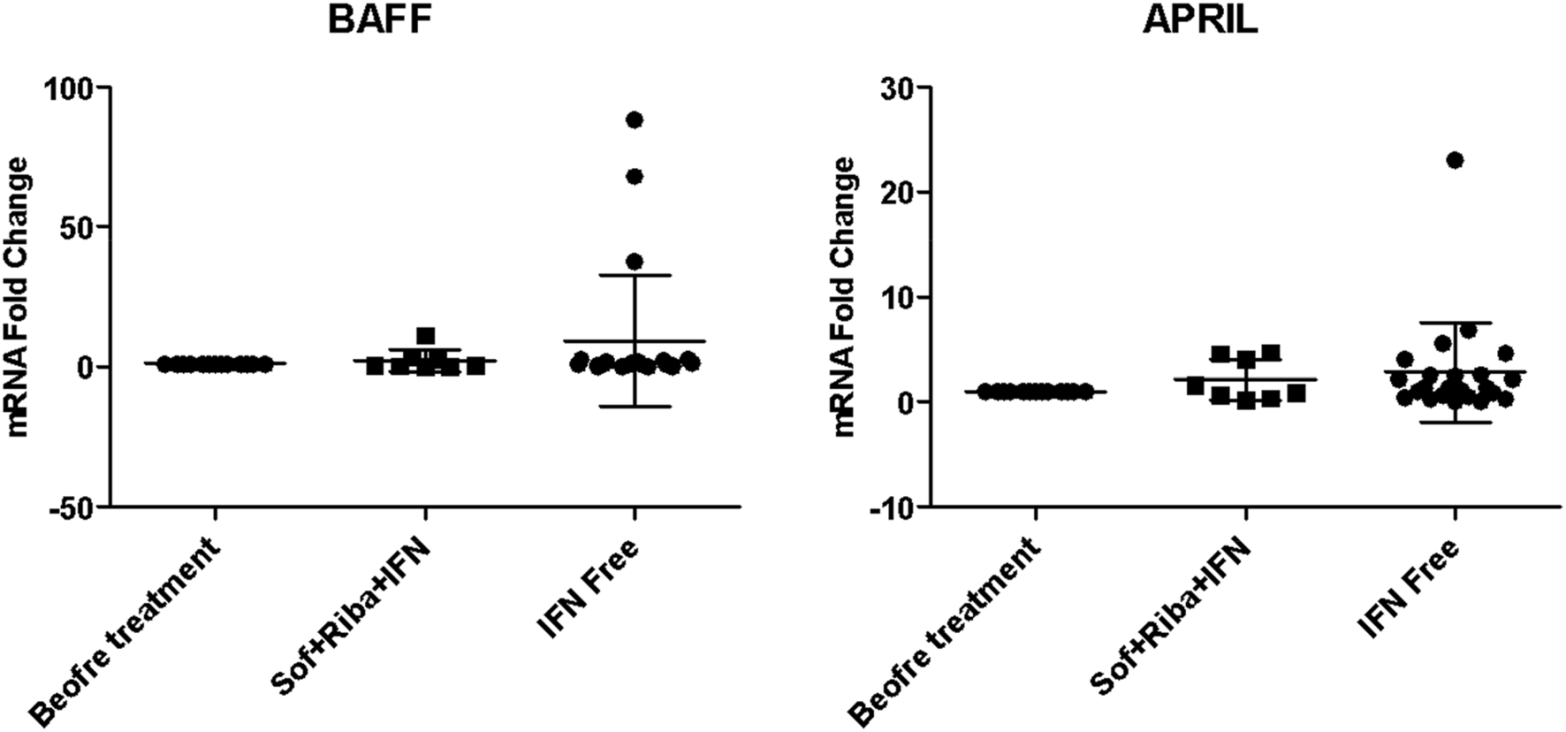
B-cells activation markers in IFN-based and IFN-free protocols.

No differences in either DNA expression profile pattern (Fig. 5) or B-cell activation markers (Fig. 6) were found between IFN-based or free regimens.

#### DNA-damage accumulation

**Figure 7:**
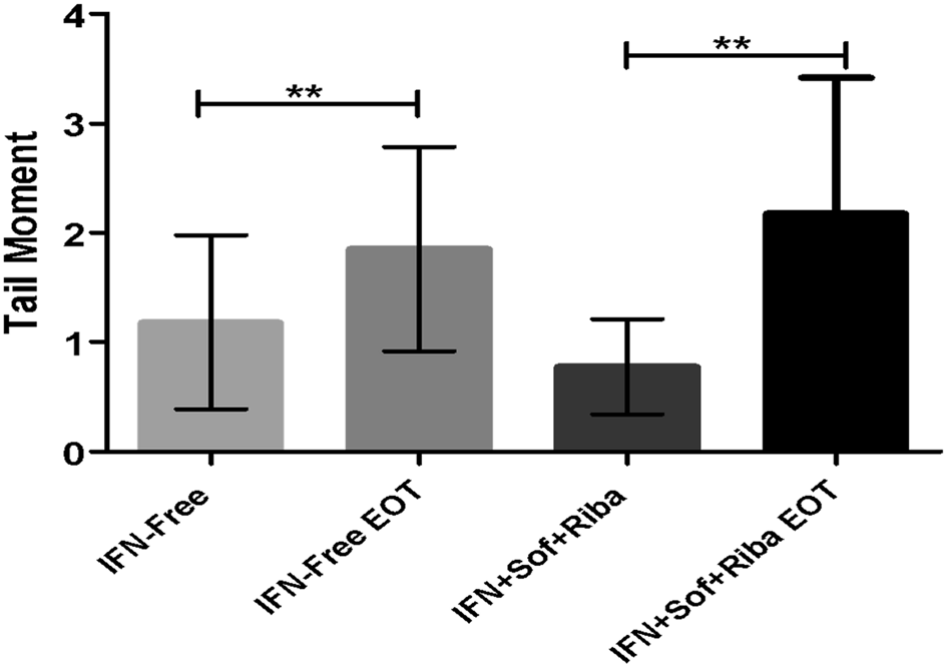
DNA-damage accumulation in IFN-based and IFN-free protocols.

A significant elevation in DNA damage levels was identified in both IFN-based and free protocols, indicating that IFN per se does not induce increased DNA damage (Fig. 7).

Of note, there were also no differences in clinical, laboratory response, relapse, DNA damage accumulation, DNA repair genes, or B cell activation markers between short (12 wk) and longer (24 wk) treatment protocols “data were not shown”.

## Discussion

Our objective in the current study was to observe HCV–MCV parameters during the course of treatment by different protocols.

To the best of our knowledge, this is the only study that reported DNA damage and repair as evidence of genomic instability in its evolution during treatment.

We were motivated by the fact that HCV infection and HCV–MCV were associated with an increased risk of malignancy (Hepatocellular carcinoma and B-cell lymphoma). We therefore included this objective in our study.

Also, following the era of introducing the DAAs, there are many reports on hepatocellular and hematological malignancies^4–7^.

Scheifer and his group reported that acute myeloid leukemia seemed more frequent after using DAA treatments, notably in severe HCV patients including cirrhotic and/or liver-transplanted patients^6^.

In a recent study by Cacoub and his group on the impact of DAAs on B cells, HCV has been shown to induce many B-cell lymphoproliferative disorders ranging from hypergammaglobulinaemia and mixed cryoglobulinemia to marginal zone lymphoma and B-Non-Hodgkin lymphoma. The exact underlying mechanism that leads from one extreme to another is not fully understood, especially the final steps before overt malignancy^11^.

Colantuono and his colleagues concluded that there is a need for a better understanding of the mechanisms of HCV–MCV resistance to the cure by DAAs and strategies have to be developed to better identify patients at risk and to prevent dangerous relapses^12^.

We believe that our findings can shed more light on the phenomenon by offering one of these mechanisms. Our work may simply be providing a clue in this puzzle.

DAAs are currently widely used in the treatment of hepatic and extrahepatic HCV manifestations including MCV^25^. In the study by Lauletta and colleagues, DAAs were associated with remarkably higher response rates than those previously achieved with p-IFN^26^.

Some reports showed both high SVR and clinical remission in about 78% of patients^27–29^. Others reported an overall good clinical response (88%) despite widely variable viral response rates ranging from 36 to 64% according to viral genotype^30^. There were also reports on the persistence of symptoms despite good SVR^31,32^.

In the current study, no significant clinical or laboratory response differences could be identified among the 3 different treatment protocols including the IFN-based regimen. Despite achieving SVR, some patients expressed relapse of articular (5/17) and constitutional manifestations (5/19) as well as CGs (5/20) also regardless of their treatment protocol.

Surprisingly, none of our patients who were treated with IFN-based protocol had significant clinico-laboratory relapse compared to those who received IFN-free treatment who showed statistically significant constitutional manifestations relapse (*P* = 0.044) and cryocrit % relapse, though non-significant (*P* = 0.07). The observed relapse rate in our DAAs treated patients is actually in agreement with one study where patients treated with DAAs were associated with significantly lower reduction of CGs, circulating B-cell clones, and higher MCV relapses, the authors attributed this to the superiority of IFN to DAAs on improving clinico-immunological outcomes^12^. This can be explained by the antiproliferative activity of IFN that could halt B-cell clonal expansion in addition to eradicating HCV and preventing B-cell antigenic stimulation^4,33^.

Two studies showed relapse characteristics resembling those seen in our patients with joint, constitutional, and cryocrit relapses that occurred early in the first year after antiviral discontinuation despite persistent SVR. However, unlike our results, relapse in their study occurred in IFN-based therapy^31,34^.

Recently, an international multicenter study for long-term follow-up of cases with HCV–MCV showed a relapse rate of 12.6% with male sex, skin ulceration, kidney involvement at baseline, and peripheral neuropathy at the end of DAA treatment being the baseline risk factors associated with higher relapse rate^35^. The inconsistent findings of relapses in HCV–MCV patients receiving DAAs therapies whether with or without IFN can be explained by the variable timing onset of initiation of antiviral therapy regardless of the protocol used and the limited number of patients investigated in these studies, making it difficult to make a solid conclusion. Other factors that might have contributed to this inconsistency include ethnicity, environmental factors, and/or HCV genotype. All our patients were Egyptian residents of the Cairo Metropolitan area (they all receive treatment according to their geographical location and they were all from the same socioeconomic pool). They were all of the Genotype IV as registered in their files.

HCV core proteins suppress DNA repair capacity^36^, whereas the HCV E2-CD81 interaction induces double-stranded DNA breaks^37^ and the HCV NS5A protein induces chromosome instability^38^.

Serum levels of BAFF and APRIL were higher in patients with autoimmune diseases like SLE, primary Sjögren's disease, and RA compared to healthy individuals^39,40^. BAFF may contribute also to the production of CGs in chronic HCV infection^41,42^.

In the current study, we found no significant differences between IFN-based and free regimens or between shorter and longer treatment protocols regarding DNA damage, markers of DNA repair, or B cell activators including BAFF and APRIL. A significant elevation in DNA damage levels was identified in both IFN-based and free protocols, indicating that IFN per se does not induce increased DNA damage. However, an individualized drug-to-drug comparisons showed many differences. Those who were treated with IFN-based protocol showed decreased levels of DNA damage at 24 weeks that totally normalized at 48 weeks to its pretreatment levels. On the other hand, BAFF levels continued to increase significantly throughout the follow-up period. In group 2 patients, CGs relapsed significantly during the follow-up period at 48 weeks with increased DNA damage levels at EOT that decreased but did not normalize as IFN-based did during the follow-up period. Sera from these patients showed increased levels of BAFF and APRIL but not significantly as did IFN-based therapy at both EOT and follow-up period. Patients treated with arm 3 had the best BAFF and APRIL response and in contrast the worst DNA damage response. BAFF and APRIL levels increased at EOT as did the other 2 protocols but decreased at 24 weeks. On the other hand, DNA damage increased at EOT and unlike the other 2 protocols remained increased at the 24-week follow-up period. Unfortunately, patients in this group couldn’t be followed up to 48 weeks. Our study showed almost overall recruitment of DNA repair markers over time with no significant differences neither between IFN-based and free nor between shorter and longer durations protocols. However, individualized head-to-head drug comparisons showed a slightly preferable profile in arm 3 protocol “taking into consideration the unavailability of longer follow-up visit at 48 weeks in this group”. Patients among this group had lower TOP1 and TOPA2 levels at 24-week follow-up visits compared to their pretreatment levels though non-significant. Our results were consistent with previous findings which showed that topoisomerases were implicated in several immunological diseases^43^ and that high titer TOP1 autoimmune antibodies are common among patients with autoimmune diseases^44,45^ and carry a poor prognosis and high mortality rates^45,46^.

Inhibition of Top2 stimulates IFN-α/β production which seems to have an antiviral activity^47^. Moreover, TOP1 has been shown to promote transcriptional progression independent of its topoisomerase activity^48^.

Group 3 treated patients in our study showed a significant reduction of poly(ADP-ribose) polymerase-1 (PARP1) at 24 weeks compared to their pretreatment levels. PARP1 is a transcription regulator of proinflammatory cytokines and chemokines. It was previously reported to have a role in RA and its inhibition reduces disease severity^49,50^. As a result of DNA damage, PARP1 is constantly activated leading to NAD + and ATP depletion with consequent cell energy failure and cell death^51^. PARP1, PARP2, and the molecular scaffold protein XRCC1 are known nowadays to accelerate DNA base excision repair (BER)^52–55^.

Hereditary mutations in XRCC1 were shown in neurodevelopmental disorders and/or progressive neurodegeneration^56^.

In the current work, TDP1 significantly decreased in the sera of group 3 patients at EOT with a more significant reduction at 24-week follow-up. Impaired TDP1 renders the cells more susceptible to single-strand break-inducing agents^57^ and defects of human TDP1 result in spinocerebellar ataxia with axonal neuropathy^58^, and recently has been implicated in repairing DNA damage at gene regulatory regions^59^.

Whether SOF or its combinations, interacts/inhibits the TDP1 and leads to accumulating irreparable DSBs is yet to be investigated.

Finally, we think that multiple factors other than SVR have to be taken into consideration when treating HCV–MCV. We suggest further research in the field of “anti-HCV drugs” induced DNA damage, breaks, and B cell activating markers. Treatment failure and/or relapse noticed in HCV–MCV patients may be related to the fact that inflammation may be a result of uncontrolled DNA repair activities or accumulating DNA damage due to impaired DNA repair^60^. The immune system will be stimulated with impairment of the normal balance between DNA damage and repair processes via multiple mechanisms including induction of stimulator of interferon genes pathway and the production of type I IFN.

The role of BAFF and APRIL in HCV–MCV and in lymphoproliferation and the need of immunosuppression in those patients may suggest a future role for anti-BlyS therapies in the management of cases with MCV beside antiviral therapy.

Limitations of our study include the small number of patients in each treatment group which was governed by available protocols at the Egyptian MOH during the study period. We could not also continue following up all patients for 48 weeks especially those in the third group who started therapy lately according to drug availability at MOH.

We are still in need of randomized controlled trials with larger numbers of patients and more importantly longer duration of follow-up, especially for DAAs-based regimens. We also need a better understanding of the mechanisms beyond viral triggering of the immune response, the behavior of immune cells before, during, and after antiviral treatment, the role of the host genetic factors, and DNA strand breaks and repair.

## Conclusion

Our study supports previous data suggesting that IFN-based therapy in patients with HCV–MCV is not only as effective as DAAs but may have the advantage of lesser relapse rates, and decreased levels of DNA damage.

Our findings encourage revisiting IFN possibly finding a niche to reconsider its use in the armamentarium of HCV-related complications, but this necessitates further investigations with larger numbers of patients for longer duration of follow-up.

We believe that our findings can shed more light and can offer an explanation for lymphoproliferation and the development of malignancies as well as relapses by suggesting one of these mechanisms. Our work may simply be providing a clue in this puzzle.

### Supplementary Information

Supplementary Table 1.

## Data Availability

All data generated or analyzed during this study are included in this published article [and its supplementary information files].

## Abbreviations

ACR: American college of rheumatology (ACR)
APRIL: A proliferation-inducing ligand
BAFF: B-cell activating factor
C4: Complement 4
CGs: Cryoglobulins
CR: Complete response
CV: Cryoglobulinemic vasculitis
DAAs: Directly acting antiviral drugs
DACLA: Daclatasvir
EOT: End of treatment
HCC: Hepatocellular carcinoma
HCV: Hepatitis C virus
MCV: Mixed cryoglobulinemic vasculitis
MOH: Egyptian ministry of health
NR: Non-responders
pIFN α: Pegylated interferon α
PR: Partial response
RBV: Ribavirin
RF: Rheumatoid factor
SLE: Systemic lupus erythematosus
SOF: Sofosbuvir
Wk: Week
