# Supplementary material for: A comparative study of different antiviral treatment protocols in HCV related cryoglobulinemic vasculitis
Source: Sci Rep. 2024 May 23;14:11840. doi: 10.1038/s41598-024-60490-z (PMC11116471; doi:10.1038/s41598-024-60490-z)
Supplement: Supplementary file 1 — Supplementary Table 1. [file 41598_2024_60490_MOESM1_ESM.docx]

**Supplementary table 1**:

**Relapses at one year follow-up comparing to EOT:**

|  |  | | | | **end of treatment JOINT** | | | | | | |  |
| --- | --- | --- | --- | --- | --- | --- | --- | --- | --- | --- | --- | --- |
|  |  | | | | **no response** | | | | **response** | | | **P value** |
|  |  | | | | **Count** | | **%** | | **Count** | | **%** |  |
| **JOINT D** | **deterioration** | | | | ***3*** | | ***75.0%*** | | ***5*** | | ***29.4%*** | ***0.009*** |
|  | **no response** | | | | ***1*** | | ***25.0%*** | | ***0*** | | ***.0%*** |  |
|  | **response** | | | | ***0*** | | ***.0%*** | | ***12*** | | ***70.6%*** |  |
|  | |  | | | **end of treatment Neuropathy** | | | | | | |  |
| **Neuropathy D** | | **deterioration** | | | ***2*** | ***50.0%*** | | ***6*** | | ***40.0%*** | | ***1*** |
|  |  | **no response** | | | ***0*** | ***.0%*** | | ***0*** | | ***.0%*** | |  |
|  |  | **response** | | | ***2*** | ***50.0%*** | | ***9*** | | ***60.0%*** | |  |
|  | | |  | | **end of treatment constitutional** | | | | | | |  |
| **constitutional D** | | | **deterioration** | | ***3*** | ***75.0%*** | | ***5*** | | ***26.3%*** | | ***0.006*** |
|  |  |  | **no response** | | ***1*** | ***25.0%*** | | ***0*** | | ***.0%*** | |  |
|  |  |  | **response** | | ***0*** | ***.0%*** | | ***14*** | | ***73.7%*** | |  |
|  | | | |  | **end of treatment Rheumatoid Factor IU/ml response** | | | | | | |  |
| **Rheumatoid Factor IU/ml D response** | | | | **deterioration** | ***1*** | ***20.0%*** | | ***5*** | | ***27.8%*** | | ***1*** |
|  |  |  |  | **no response** | ***0*** | ***.0%*** | | ***0*** | | ***.0%*** | |  |
|  |  |  |  | **response** | ***4*** | ***80.0%*** | | ***13*** | | ***72.2%*** | |  |
|  | | | |  | **end of treatment Complement C4 mg/dl response** | | | | | | |  |
| **Complement C4 mg/dl D response** | | | | **deterioration** | ***2*** | ***33.3%*** | | ***2*** | | ***11.8%*** | | ***0.270*** |
|  |  |  |  | **no response** | ***0*** | ***.0%*** | | ***0*** | | ***.0%*** | |  |
|  |  |  |  | **response** | ***4*** | ***66.7%*** | | ***15*** | | ***88.2%*** | |  |
|  | | | |  | **end of treatment CRYO % response** | | | | | | |  |
| **CRYO % D response** | | | | **deterioration** | ***0*** | ***.0%*** | | ***5*** | | ***25.0%*** | | ***0.002*** |
|  |  |  |  | **no response** | ***3*** | ***100.0%*** | | ***1*** | | ***5.0%*** | |  |
|  |  |  |  | **response** | ***0*** | ***.0%*** | | ***14*** | | ***70.0%*** | |  |
